# Correction: Relationship between HIV viral suppression and multidrug resistant tuberculosis treatment outcomes

**DOI:** 10.1371/journal.pgph.0004337

**Published:** 2025-02-26

**Keri Geiger, Amita Patil, Chakra Budhathoki, Kelly E. Dooley, Kelly Lowensen, Norbert Ndjeka, Jacqueline Ngozo, Jason E. Farley**

In the Abstract section, there is an error in the sixth sentence. The correct sentence is: Those who became detectable or were never suppressed were at significantly increased risk of death (RRR 11.50, 95% CI 1.98-66.65; RRR 9.28, 95% CI 1.53-56.61), treatment failure (RRR 6.37, 95% CI 1.58-25.70; RRR 4.54, 95% CI 1.35-15.24), and loss to follow-up (RRR 7.00, 95% CI 2.83–17.31; RRR 2.97, 95% CI 1.02–8.61) compared to those who maintained viral suppression.

Tables 1 to 4 are uploaded incorrectly. Please see the correct Tables 1 to 4 here.

**Table 1:** Demographic and Clinical Characteristics of PWH (n=1479).

		Total	HIV Viral Load Status at MDR-TB Treatment Initiation		
			Virally Suppressed	Detectable Viral Load	Viral Load Unknown
Total		1479 (100.0%)	457 (30.9%)	524 (35.4%)	498 (33.7%)
Age, mean (SD, IQR)		37.1 (10.3, 30-43)	39.1 (10.5, 31-46)	36.1 (9.7, 29-42)	36.5 (10.5, 29-42)
Sex	Male	809 (54.7%)	226 (49.5%)	298 (56.9%)	285 (57.2%)
	Female	670 (45.3%)	231 (50.6%)	226 (43.1%)	213 (42.8%)
Baseline CD4 Count, median (IQR)		182 (75-363)	385.5 (193-543)	164.2 (44.5-273.5)	222.1 (66.5-321.5)
Baseline BMI, median (IQR)		20.7 (17.5-22.8)	21.1 (17.6-23.4)	20.2 (17.5-21.8)	20.8 (17.4-23.2)
Prior TB Episodes	None	649 (43.9%)	177 (38.7%)	213 (40.7%)	259 (52.0%)
	One	657 (44.4%)	225 (49.2%)	257 (49.1%)	175 (35.1%)
	Two or More	117 (7.9%)	38 (8.3%)	43 (8.2%)	36 (7.2%)
	Unknown	56 (3.8%)	17 (3.7%)	11 (2.1%)	28 (5.6%)
ART Status at MDR-TB Treatment Initiation	Taking ART	887 (60.0%)	421 (92.1%)	266 (50.8%)	200 (40.2%)
	Not Taking ART	592 (40.0%)	36 (7.9%)	258 (49.2%)	298 (59.8%)
MDR-TB Regimen	Injectable	808 (54.6%)	248 (54.3%)	287 (54.8%)	273 (54.8%)
	All-oral	386 (26.1%)	117 (25.6%)	151 (28.8%)	118 (23.7%)
	Both	254 (17.2%)	86 (18.9%)	76 (14.5%)	92 (18.5%)
	Individualized	31 (2.1%)	6 (1.3%)	10 (1.9%)	15 (3.0%)
MDR-TB Outcome	Treatment Success	925 (62.5%)	327 (71.6%)	293 (55.9%)	305 (61.2%)
	Death	212 (14.3%)	11 (2.4%)	19 (3.6%)	18 (3.6%)
	Treatment Failure	48 (3.3%)	38 (8.3%)	103 (19.7%)	71 (14.3%)
	Lost to Follow-Up	294 (19.9%)	81 (17.7%)	109 (20.8%)	104 (20.8%)

PWH, people with HIV; n, number; HIV, human immunodeficiency virus; TB, tuberculosis; MDR-TB, multidrug-resistant TB; SD, standard deviation; IQR, interquartile range; BMI, body mass index; ART, antiretroviral therapy. Note: Some percentages do not add to 100.0% due to rounding.

**Table 2:** Comparison of Negative Outcomes Among PWH With and Without HIV Viral Suppression at MDR-TB Treatment Initiation Using a Multinomial Model (n=1479).

Baseline HIV Viral Load (ref. Undetectable)	MDR-TB Treatment Outcome					
	Died		Treatment Failure		Lost to Follow-Up	
	RRRˆ	95% CI	RRRˆ	95% CI	RRR	95% CI
Detectable	2.12^*^	1.11-4.07	1.72	0.96-3.05	1.39	0.92-2.08
Unknown	1.64	0.85-3.16	1.64	0.91-2.94	1.28	0.80-2.04

MDR-TB, multidrug-resistant tuberculosis; HIV, human immunodeficiency virus; ref., reference; RRR, relative risk ratio; CI, confidence interval; *, p<0.05; **, p<0.01; ***, p<0.001. **ˆ**Note: adjusted for age, sex, baseline CD4 count, and baseline BMI.

**Table 3:** MDR-TB Treatment Outcome by HIV Viral Load Status Over Time (n=673).

HIV Viral Load Status Over Time	Total	MDR-TB Treatment Outcome, n (%)			
		Success	Died	Failure	Lost to Follow-Up
Total	673 (100.0%)	537 (79.8%)	46 (6.8%)	23 (3.4%)	67 (10.0%)
Maintained Suppression	194 (100.0%)	176 (90.7%)	4 (2.1%)	4 (2.1%)	10 (5.2%)
Became Suppressed	267 (100.0%)	223 (83.5%)	15 (5.6%)	8 (3.0%)	21 (7.9%)
Became Detectable	94 (100.0%)	53 (56.4%)	13 (13.8%)	5 (5.3%)	23 (24.5%)
Never Suppressed	118 (100.0%)	85 (72.0%)	14 (11.9%)	6 (5.1%)	13 (11.0%)

MDR-TB, multidrug-resistant tuberculosis; HIV, human immunodeficiency virus; n, number. Note: Some percentages do not add to 100.0% due to rounding.

**Table 4:** Comparison of Negative Outcomes Among PWH According to HIV Viral Load Changes Between MDR-TB Treatment Initiation and MDR-TB Treatment Outcome Using a Multinomial Model (n=673).

HIV Viral Load Status Over Time (ref. Maintained Suppression)	MDR-TB Treatment Outcome					
	Died		Treatment Failure		Lost to Follow-Up	
	RRRˆ	95% CI	RRRˆ	95% CI	RRRˆ	95% CI
Became Suppressed	2.75	0.66-11.42	1.53	0.83-2.84	1.22	0.40-3.76
Became Detectable	11.50**	1.98-66.65	6.37**	1.58-25.70	7.00***	2.83-17.31
Never Suppressed	9.28*	1.52-56.61	4.54*	1.35-15.24	2.97*	1.02-8.61

MDR-TB, multidrug-resistant tuberculosis; HIV, human immunodeficiency virus; ref., reference category; RRR, relative risk ratio; CI, conficence interval; *, p<0.05; **, p<0.01; ***, p<0.001. **ˆ**Note: adjusted for age, sex, length of MDR-TB treatment, MDR-TB regimen, and arm of parent study.
