# Supplementary material for: Relationship between HIV viral suppression and multidrug resistant tuberculosis treatment outcomes
Source: PLOS Glob Public Health. 2024 May 6;4(5):e0002714. doi: 10.1371/journal.pgph.0002714 (PMC11073678; doi:10.1371/journal.pgph.0002714)
Supplement: Attachment — Submitted filename: Response to Reviewers_2.docx [file pgph.0002714.s002.docx]

December 13, 2023

Editorial Team

PLOS Global Public Health

Dear Dr Editorial Team,

Thank you for your ongoing consideration of this manuscript. Per your instructions, all data have been provided by uploading the files as Supplementary Information. Please note that Stata version 16 was used for this analysis, and the Stata .do file containing the script, the raw data as an Excel file, and the final data as a .dta file are included in supplemental information. These are the only data files used in this analysis.

Thank you and I look forward to hearing from you again soon.

Sincerely,

Keri Geiger, PhD, RN, BSN, ACRN

Johns Hopkins University School of Nursing

525 North Wolfe Street

Baltimore, MD 21205

(804) 774-6774

klgeiger191@gmail.com
